# Understanding the Interaction between Regulatory Focus and Message Framing in Determining Chinese Consumers’ Attitudes toward Artificial Meat

**DOI:** 10.3390/ijerph19094948

**Published:** 2022-04-19

**Authors:** Hongxu Shi, Peihua Ma, Yinchu Zeng, Jiping Sheng

**Affiliations:** 1 School of Agricultural Economics and Rural Development, Renmin University of China, Beijing 100872, China; shihongxu@ruc.edu.cn (H.S.); zengyc@ruc.edu.cn (Y.Z.); 2 Department of Nutrition and Food Science, College of Agriculture and Natural Resources, University of Maryland, College Park, MD 20740, USA; peihua@umd.edu

**Keywords:** artificial meat, consumer attitude, regulatory focus, structural equation modeling, moderating effect

## Abstract

While production and consumption of meat cast a shadow over the prospects for sustainable development, artificial meat may be the solution. However, consumer acceptability of artificial meat is a major impediment to its use as a suitable alternative. This study analyzed the relationship between regulatory focus and consumer acceptance of artificial meat using randomized controlled trial data. Results showed that promotion focus results in a higher acceptance of artificial meat products due to a higher perceived benefit and lower perceived risk, whereas prevention focus results in a lower acceptance of artificial meat products due to perceived benefit being lower and perceived risk being higher. The moderating effect of the message framing was investigated employing structural equation modeling (SEM). It was discovered that a gain-oriented message framing could greatly strengthen the association between promotion focus and perceived benefit, whereas an avoidance-oriented message framing could significantly diminish the relationship between prevention focus and perceived risk. This study has crucial implications for how policymakers and industries communicate with consumers about artificial meat.

## 1. Introduction

The global community is at a critical moment in its pursuit of Seventeen Sustainable Development Goals (SDGs). SDGs have directed many public initiatives, company policies, educational efforts, research activities, etc. Ensuring food security, avoiding wasting water, promote energy efficiency, and stop global warming (SDG:2;6;7;13) with regard to what we eat—less animal-based and more plant-based—and how food is produced [1,2]. The concerns about environmental degradation, public health, and animal welfare caused by the production and consumption of traditional meat have attracted extensive attention. Traditional meat consumption has a negative impact on global food security and consumes large amounts of resources. Thirty-five percent of the crops produced by agricultural systems are consumed by animals [3]. In total, 70% of global water consumption and 20% of energy consumption are directly or indirectly used in the production of animal products [4]. GHG emissions from the production of animal products exceed those emitted by all modes of transportation combined [5]. Substituting artificial meat for traditional meat is an important way to achieve the Sustainable Development Goals and has received a lot of attention from existing research. Artificial meat, including plant-based meat and cell-based meat, represents changes to current food systems that can reduce GHG emissions, water input, energy input, and ensure food security [6,7,8,9,10,11,12,13].

As a young industry, artificial meat is in constant evolution, seeking ways to overcome three key challenges: standardization, environmental sustainability, and profitability [14]. Another critical challenge is that consumers are skeptical of artificial meat [15,16]. It is therefore important to understand consumers’ perceptions of artificial meat, including those aspects they consider favorable and unfavorable.

A body of consumer insights regarding artificial meat and related novel technologies for food production already exists. In various ways, it illuminates the mix of potential advantages and disadvantages of artificial meat to consumers, including improved security in food supply, less GHG emissions, good for human health, good for animal welfare, and less antibiotic use (advantages), but premium pricing, concerns over the decline of traditional agriculture, and concerns over bad tastes (disadvantages). Stakeholder insights have also been keenly sought, often through depth interviews and/or case studies [13,16,17,18,19,20,21]. A shortcoming of this growing literature is the lack of exploration of attitudes toward and perceptions of artificial meat from different message framing settings in China. Such undertakings are pertinent considering that China is the largest consumer of meat. In this regard, consumer attitudes in China are particularly relevant because of the region’s population density and its many megacities.

This study adds to the existing body of knowledge in three ways. To begin with, the current research adds new consumer insights by conducting a randomized controlled trial investigation in which respondents were exposed to various message framing describing artificial meat in order to ascertain which aspects consumers find acceptable or unacceptable. Consumer attitudes were elicited directly in response to the researcher’s message framing of artificial meat—that is, gain-oriented message framing/avoidance-oriented message framing. The gain-oriented message emphasizes the benefits of artificial meat, while the avoidance-oriented message emphasizes the risks of artificial meat. The messages presented in the control and treatment groups are based on the companies MOSAMEAT and IMPOSSIBLEFOOD’s descriptions of artificial meat. While numerous previous studies have examined the effects of message framing and regulatory focus, the concentration of artificial meat products is relatively low. Second, this study empirically estimates and compares consumers’ acceptance across different regulatory focus and information types, and further reveals the effects of information and regulatory focus on attitude. Third, this study analyzes nationally representative data from China in order to shed light not only on consumer attitudes toward artificial meat, but also on the extent to which those attitudes are influenced by message framing, thereby filling a knowledge gap in Asia. This will help manufacturers use the information framework to target specific user groups to promote artificial meat products.

## 2. Literature

### 2.1. Message Framing and Regulatory Focus Theory

Attitudes can be thought of as affective associations that predispose consumers to evaluate a particular concept as positive or negative [22,23], and measuring attitudes is critical for comprehending consumer behavior [22]. The most frequently used method of measuring attitudes toward specific issues is to rely on explicit measures of participants’ responses to Likert-type scales [23], which may include asking participants to rate their level of agreement with a series of statements. Due to consumers’ limited knowledge of the artificial meat production process and nutrition in relation to novel food products, it is impractical to study consumer attitudes toward artificial meat without providing any message about it. However, the message framing used to describe artificial meat may have a significant impact on consumer attitudes toward novel food products [24,25], and it is critical for industry and policymakers to gain a comprehensive understanding of the message framing effects on artificial meat products.

The term “message framing” refers to a linguistic presentation strategy used to increase the persuasiveness of a particular message. The effect of message framing is theoretically based on Kahneman and Tversky’s prospect theory, which asserts that consumers’ value functions in decision-making are classified as gain or loss [26]. Consumers confronted with risky outcomes may, according to prospect theory, evaluate gains and losses differently, and their preferences may vary in terms of negative or positive framing. Previous research indicated that the way information or a message is presented has a significant impact on consumers’ purchasing decisions [24,27,28,29]. However, few studies have examined the effect of message framing on consumer perceptions of artificial meat.

Additionally, the impact of message framing on consumers may be highly varied, since individuals tend to concentrate only on the message that interests them. It has been hypothesized that the regulatory focus acts as a filter for information, causing people to react differently to the same message [30]. As a result, it is equally critical to grasp the regulatory focus. Higgins advances the regulatory focus hypothesis [31], which establishes a distinction between two systems that govern people’s judgments and actions. A promotion focus (Pro) stresses the pursuit of gains and ambitions toward an ideal, which develops an enthusiastic approach mentality. By contrast, a prevention focus (Pre) places a premium on avoiding loss and meeting responsibilities, which promotes a watchful attitude of avoidance [31]. Existing research indicates that when messages are framed in a way that corresponds to the consumer’s regulatory priorities, they are more compelling [32,33]. It has been proposed that when an individual’s regulatory focus and message framing align, his or her value judgment on information is enhanced, hence boosting the individual’s subjective appraisal of a product [34]. However, there is a dearth of studies examining consumer attitudes toward artificial meat using the regulatory focus theory, particularly in Asia.

### 2.2. Perceived Benefits

The term “perceived benefits” refers to “the perceived net advantages connected with the purchased items or services” [35]. Consumers assess the advantages they believe they will achieve from obtaining and utilizing the product/service against the dangers associated with doing so [35]. Frequently, this “benefit” component is derived from the product/quality of service or the purchasers’ perception of the product/quality of service [35]. The idea of perceived advantage is commonly used to explain why a person engages in a certain activity or action [36]. Both functional and non-functional motives impact the human determinants of consumption behavior [37]. Functional motivations are those that are associated with utilitarian functions such as convenience, performance, physical, and financial rewards, while non-functional reasons are those that are associated with social and emotional requirements [36,38]. Utilitarian advantages are essentially instrumental, functional, and cognitive in nature; they give clients value by serving as a means to an aim. Hedonic advantages are non-instrumental, experiential, emotional, and emotive; they are appreciated independently of their practical utility [38,39].

Similarly, customers’ benefits from artificial meat might be classified as utilitarian or hedonic. Consumers benefit from utilitarian features that increase the usefulness, efficiency, and economy of their purchases. For instance, consider the safety and nutritional value of artificial meat. In addition, the social and psychological benefits of artificial meat can be classified as hedonic, because they provide intrinsic pleasure, fun, and self-esteem. For instance, the environmental and animal welfare benefits of fake meat are a social acquisition with positive externalities, and this emotion is critical to achieving the goal of artificial meat promotion. Additionally, because regulatory focus is connected to perceived benefits and perceived benefits are related to consumer behavior [40], this study explores the function of perceived benefits as a significant intermediary variable in the relationship between regulatory focus and consumer attitudes.

### 2.3. Perceived Risks

The term “perceived risks” refers to a consumer’s subjective assessment of the inherent hazards associated with achieving a desired objective [41]. Consumers’ behaviors create an atmosphere of risk, as the probable adverse repercussions of their actions cannot be predicted with confidence [42]. The term “perceived risk” refers to the sum of the negative repercussions of a loss and the probability of bad outcomes [42,43,44]. In other words, perceived risk is defined as the difference between a customer’s pre-buy uncertainty about the kind and extent of anticipated loss associated with the purchase and use of a product and the actual risk associated with the purchase and use of the product [45]. In comparison to conventional meat, the function of perceived risk is more significant when it comes to artificial meat, owing to the increased uncertainty and unpredictability. Artificial meat provides very novel items and services. There is a scarcity of high-quality information on artificial meat, which increases perceived risk. As a result, artificial meat includes more variable components that cannot be checked thoroughly before purchase, making it harder to adequately assess risks.

Prior research has identified numerous characteristics of perceived risk that are significant in the context of consumption, including performance risk, financial risk, psychological risk, social risk, and physical danger [46,47]. Physical risk is concerned with the safety of commodities and the possible dangers or damages connected with their acquisition or usage [48]. Safety concerns pertain to consumers’ perceived sense of safety and security as a result of service providers emphasizing the emotional relief of customers who may be concerned about issues such as danger, injury, or loss [37]. Psychological risk is associated with the possibility of developing a negative self-image or self-concept as a result of the purchase or usage of goods [37]. Increased unpredictability in artificial meat products leads to an increased level of risk for the consumer. The relationship between perceived risk and moderated focus is often emphasized [40], and as perceived risk is closely related to consumer behavioral attitudes, this study will examine the important role of perceived risk as an intermediate variable.

### 2.4. Variables for Balance Check

Consumption of food is a behavior that is influenced by a variety of factors, including beliefs and habits. The primary objective of this study was to determine the effect of message framing on consumer perceptions of artificial meat. Thus, prior to conducting the study, it was necessary to ascertain that there were no significant differences in the sample’s food consumption beliefs and practices and to eliminate potential confounding variables, i.e., to conduct a balance test. By combining the study’s actual situation with the available literature, we identified three major categories of variables for the balance test: food-related lifestyle, food novelty fear, and food technology support attitude.

Food technologies generate a significant level of perceived risk since they impact important consumer concerns such as food safety, animal welfare, and the environment [49,50]. Thus, the amount of food innovation is crucial to customer approval, and consumer acceptance of new foods is influenced by both the kind of innovation and the product that incorporates it [51]. The majority of prior research on food innovation and consumption has concentrated on food neophobia [52]. Therefore, we used food neophobia as one of the variables for the balance test. Additionally, the majority of research on consumer acceptability of revolutionary food technologies has been undertaken in chosen European or North American nations, with a dearth of studies from Asian countries [53]. Consumption patterns of traditional meats by consumers are also a significant background component, which is why we include them [11,54]. In addition, consumer attitudes toward food technology may also be a variable that cannot be ignored [17].

Specifically, four questions are used to assess respondents’ level of food neophobia, which refers to an individual’s aversion to unfamiliar foods. A higher score on each of the food neophobia questions indicates that the responder is more hesitant to try unusual foods [55]. Five questions are aimed at ascertaining respondents’ level of food involvement, which relates to an individual’s efforts associated with the food they consume. A higher degree of food involvement (as measured by a score on related items) indicates that people care about the food they consume and make an effort to prepare it [56]. Three questions assess respondents’ support for food technology, while five others assess respondents’ meat eating habits [54]. Finally, eleven questions are used to assess respondents’ meat attitudes in the second part of the survey, including topics such as meat pleasure, health, the environment, and animal welfare. For instance, the term “enjoyment associated with meat belief” relates to respondents’ satisfaction with meat products, with higher scores on related questions indicating that respondents experienced greater enjoyment from eating meat.

## 3. Materials and Methods

### 3.1. Message Design

To examine how message framing affects consumer views about artificial meat, we randomly assigned our study participants to one of three message groups with varying message framing. The first category of information is the control group, which provides merely a neutral description of artificial meat. The other two types of information are for treatment purposes. Along with the neutral description of artificial meat used in the control group, the other two treatment groups include a gain-oriented and an avoidance-oriented message regarding artificial meat, respectively. The messages associated with each of the three message categories are provided in Table A1. Similar to the randomized controlled trial (RCT) method, we randomly allocate each participant to one of three information groups: neutral, gain-, or avoidance-oriented. As a result, study participants are randomly assigned to one of three information groups. Following the reading of the information, questionnaires concerning artificial meat are answered by the participants. We developed questions to elicit customer perceptions of two types of artificial meat technologies nowadays in practice (plant-based meat and cultured meat).

### 3.2. Data and Variable

All participants self-registered with an approved web panel (online survey) provider (WenJuanXing), the biggest online survey provider in China. The survey was conducted in December 2019 and concluded in January 2020. The final sample consisted of 2226 completed questionnaires. We develop surveys to infer customers’ regulatory focus. Additionally, our research gathers data on consumers’ meat beliefs, food involvement, food neophobia, attitudes toward food technology, and views toward artificial meat products. Thus, effective utilization of nationally representative data is intended to give insight on China’s broad sentiments about artificial meat and on how to engage with customers via message framing. This research abides by all ethical standards and guarantees that each participant is informed. Participants were told that their replies would be kept secret and consented to participate voluntarily. Participants received a prize of around 20 yuan (RMB) as recompense.

In accordance with regulatory focus theory [31], we employ four 1–5 scale questions to assess respondents’ promotion and preventive focus, respectively, and score their responses. For example, when respondents respond “totally disagree”, “disagree”, “neutral”, “agree”, or “strongly agree” to the question “I will care about how to achieve success”, we assign a score of 1, 2, 3, 4, or 5 to the response if the respondent selects “totally disagree”, “disagree”, “neutral”, “agree”, or “strongly agree”. A higher score indicates that respondents are more concerned with achieving success and hence more likely to suit the promotion target.

### 3.3. Empirical Strategy

Since this study involves latent variables, one of the mainstream approaches to deal with latent variables is to use structural equation modeling. This study employs structural equation modeling (SEM) to investigate the potential mechanism of association between regulatory focus and attitudes moderated by message framing. The structural equation model is divided into two components: the latent variable measurement model and the structural model. A model for measuring latent variables in which the relationships between the latent variables and their observable indicators are represented. A structural model that encapsulates the relationships between latent variables. To assess the goodness-of-fit of our group-level SEM estimations, we used the standardized root mean square residual (SRMR) and coefficient of determination (CD). If the fit is good, then SRMR (standardized root mean squared residual) will be close to 0 and CD will be near 1.

More specifically, latent variables measurement model can be expressed as follows:

To better identify how the regulatory focus affects consumers attitude and further study the moderating effect of information frame, the following structural model is developed:

(1) $$\left[\begin{array}{c} \mathrm{Pro}1\\ \mathrm{Pro}2\\ \mathrm{Pro}3\\ \mathrm{Pro}4\\ \mathrm{Pre}1\\ \mathrm{Pre}2\\ \mathrm{Pre}3\\ \mathrm{Pre}4\\ \begin{array}{c} B1\\ B2\\ B3\\ B4\\ R1\\ R2\\ R3\\ R4\\ A1\\ A2\\ A3\\ A4 \end{array} \end{array}\right]=[\begin{array}{ccccc} 1 & 0 & 0 & 0 & 0\\ \lambda_{2,1} & 0 & 0 & 0 & 0\\ \lambda_{3,1} & 0 & 0 & 0 & 0\\ \lambda_{4,1} & 0 & 0 & 0 & 0\\ 0 & 1 & 0 & 0 & 0\\ 0 & \lambda_{6,2} & 0 & 0 & 0\\ 0 & \lambda_{7,2} & 0 & 0 & 0\\ 0 & \lambda_{8,2} & 0 & 0 & 0\\ 0 & 0 & 1 & 0 & 0\\ 0 & 0 & \lambda_{9,3} & 0 & 0\\ 0 & 0 & \lambda_{10,3} & 0 & 0\\ 0 & 0 & \lambda_{11,3} & 0 & 0\\ 0 & 0 & 0 & 1 & 0\\ 0 & 0 & 0 & \lambda_{13,4} & 0\\ 0 & 0 & 0 & \lambda_{14,4} & 0\\ 0 & 0 & 0 & \lambda_{15,4} & 0\\ 0 & 0 & 0 & 0 & 0\\ 0 & 0 & 0 & 0 & \lambda_{17,5}\\ 0 & 0 & 0 & 0 & \lambda_{19,5}\\ 0 & 0 & 0 & 0 & \lambda_{20,5} \end{array}]\left[\begin{array}{c} \mathrm{Pro}\\ \mathrm{Pre}\\ \mathrm{Benefit}\\ \mathrm{Risk}\\ \mathrm{Attitude} \end{array}\right]+\left[\begin{array}{c} \varepsilon_{1}\\ \varepsilon_{2}\\ \varepsilon_{3}\\ \varepsilon_{4}\\ \varepsilon_{5}\\ \varepsilon_{6}\\ \varepsilon_{7}\\ \varepsilon_{8}\\ \begin{array}{c} \varepsilon_{10}\\ \varepsilon_{11}\\ \varepsilon_{12}\\ \varepsilon_{13}\\ \varepsilon_{15}\\ \varepsilon_{16}\\ \varepsilon_{17}\\ \varepsilon_{18}\\ \begin{array}{c} \varepsilon_{20}\\ \varepsilon_{21}\\ \varepsilon_{22}\\ \varepsilon_{23} \end{array} \end{array} \end{array}\right]$$

(2) $$\left[\begin{array}{c} \mathrm{Attidude}\\ \mathrm{Benifit}\\ \mathrm{Risk} \end{array}\right]=\left[\begin{array}{cc} \beta_{1,1} & \beta_{1,2}\\ 0 & 0\\ 0 & 0 \end{array}\right]\left[\begin{array}{c} \mathrm{Benifit}\\ \mathrm{Risk} \end{array}\right]+\left[\begin{array}{cc} 0 & 0\\ \beta_{2,1} & \beta_{2,2}\\ \beta_{3,1} & \beta_{3,2} \end{array}\right]\left[\begin{array}{c} \mathrm{Pro}\\ \mathrm{Pre} \end{array}\right]+\left[\begin{array}{c} \varepsilon_{19}\\ \varepsilon_{9}\\ \varepsilon_{14} \end{array}\right]$$

**Figure 1:**
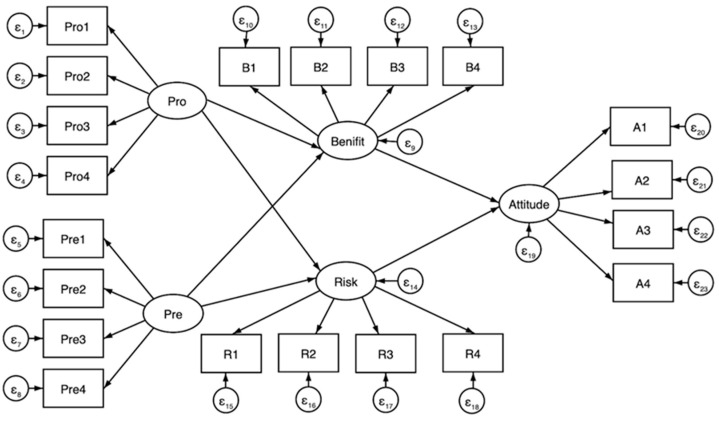
The Structural equation modeling.

The visual representation of the study model is as follows (Figure 1):

Table A3 contains the observation groups for these latent variables. Pro1–4 denotes indicators that promote focus, while Pre1–4 denotes indicators that denote prevention focus [57]. B1–4 is the perceived benefit index, whereas R1–4 is the perceived risk index. A1–4 is a proxy for consumer attitudes toward artificial meat.

## 4. Results

### 4.1. Descriptive Statistics

Table A2 shows the descriptive statistics of the descriptive demographic characteristics of the sample. The number of participants in each of the three groups was 838 (neutral message group), 692 (gain-oriented message group), and 736 (avoidance-oriented message group). Our full sample consists of 1091 male and 1175 female respondents, suggesting a balanced number of males and females. In terms of education, respondents with bachelor’s degrees account for 73.6% of the total sample. Respondents’ ages are primarily distributed between 18–40 years old, and respondents’ monthly incomes are almost evenly distributed between 2000 and 20,000.

**Figure 2:**
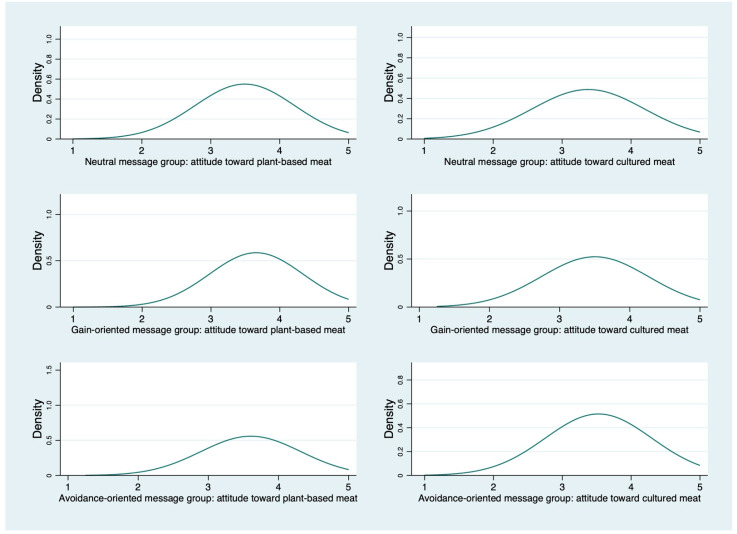
Distribution of consumer acceptance attitudes for each information group.

To obtain an initial estimate of consumer attitudes, the sum of the attitude measurement items is averaged (see Table A3). The higher the value, the more favorable consumers’ attitudes toward artificial meat products are. Figure 2 illustrates the distribution of consumer acceptance attitudes for each information group. Consumers’ attitudes toward artificial meat became more positive after information was provided (Score of plant-based meat: Neutral message group: 3.49; Gain-oriented message group: 3.65; Avoidance-oriented message group: 3.61) (Score of cultured meat: Neutral message group: 3.37; Gain-oriented message group: 3.50; Avoidance-oriented message group: 3.53).

The balance check of the sample groups entering distinct information groups is a prerequisite for the future analysis of the findings. Further study can be undertaken only if it is proven that there are no large variations in certain behaviors and beliefs regarding meat consumption between consumers in the different treatment groups before receiving the information. Certain variables are measured using multiple indicators, and their values are averaged by adding the values of the indicators used to measure them (Table A3). For example, the variable Food Technology Attitudes is measured by three indicators, and the mean score of these three measures is the value of the variable. The balance check of characteristics in Table 1 using the F test indicates that there is no significant difference between the samples of the three information groups, providing a reliable foundation for further research.

### 4.2. Results of Structural Equation Modeling

Table 2 and Table 3 show the SEM estimated results based on Equations (1) and (2), and all coefficients are statistically significant. Table 4 shows the degree of group-level goodness of fit of the structural equation model, and the results show that the overall fit is good. Promotion focus is positively related to perceived benefit and negatively related to perceived risk. Prevention focus and perceived benefit have a significant negative correlation, while prevention focus and perceived risk have a significant positive correlation. Perceived benefit was statistically significant and correlated with a positive attitude, whereas perceived risk was statistically significant and correlated with a negative attitude. As shown, promotion focus emphasis on benefits while ignoring risks, whereas prevention focuses emphasis on risks while ignoring benefits. Consumers who use promotion focus have a more positive attitude toward artificial meat by emphasizing higher perceived benefits and lower perceived risks. Because of lower perceived benefits and higher perceived risks, consumers who use prevention focus have a more negative attitude toward artificial meat.

When gain-oriented message framing is used, the relationship between stimulative focus and perceived benefit is significantly improved, as evidenced by a larger standardization coefficient (Table 2: Panel B Column 2; Table 3: Panel B Column 2). The association between prevention focus and perceived risk is significantly weakened when an avoidance-oriented message is used, specifically, as evidenced by a smaller standardization coefficient (Table 2: Panel C Column 3; Table 3: Panel C Column 3).

Additionally, the role of regulatory focus varies according to artificial meat product type. For plant-based meat, promotion focus has a greater impact on attitude (Table 2: Panel A and Table 3: Panel A), resulting in a more positive overall attitude toward plant meat. Thus, the gain-oriented message framing is more conducive to increasing people’s positive perceptions of plant meat, which in turn promotes acceptance. The public’s overall attitude toward cultured meat is more negative, owing to the fact that prevention focus plays a greater role among the regulatory focus influencing consumer intention. This is demonstrated by the increased standardized coefficient in the results (Table 2: Panel A and Table 3: Panel A). So, avoidance-oriented message framing has a more visible effect on weakening the relationship between prevention focus and risk perception, thereby assisting people with accepting cultured meat by assuaging their fears.

## 5. Conclusions

The analysis yields several key findings: Firstly, promotion focus encourages people to think positively about artificial meat by emphasizing the benefits and minimizing the concerns. The prevention focus has resulted in a higher emphasis on risks rather than benefits, decreasing the value of artificial meat. Secondly, the relationship between promotion focus and perceived benefit was strengthened by gain-oriented message framing, whereas the relationship between prevention focus and perceived risk was diminished by avoidance-oriented message framing. As a result, both sorts of the message improved people’s perceptions of artificial meat. Thirdly, for various artificial meat products, different information strategies should be used. Prevention focus has a stronger negative effect on cultured meat acceptance, and avoidance-oriented messages can weaken the link between prevention focus and perceived risk. Promotion focus has a stronger positive effect on plant meat acceptance, and gain-oriented messages can significantly strengthen the link between promotion focus and perceived gain.

The hedonic principle dominated the field of motivational psychology. It is based on the assumption that people are naturally motivated to seek pleasure and avoid pain. While the hedonic principle clearly identifies individual psychological drives or behavioral motivations, the question of how people pursue pleasure and avoid pain remains unanswered, and this is precisely what self-regulation theory is attempting to address. Regulatory focus theory, based on the hedonic principle, distinguishes the strategies and means by which individuals seek ideal states and avoid undesirable states, and specifies that different strategies and means are used for different regulatory focuses. Recent years have seen a shift toward a regulatory focus in motivational psychology research, which has been extensively used to explain individual differences in attitudes, emotions, and behaviors.

The empirical results of this study show that promotion focus has a statistically significant positive association with perceived benefits. Prevention focus has a statistically significant negative association with perceived risks. The results of this study once again validate the regulatory focus theory. According to regulatory focus theory, a gain-emergent situation activates facilitative focus, in which individuals are more sensitive to positive outcomes and actively seek to match the ideal state; a loss-emergent situation activates defensive focus, in which individuals are more sensitive to negative outcomes and actively avoid matching the undesirable state. Facilitative focus increases sensitivity to positive outcomes by utilizing a “desire” strategy approach to ensure hits against overlooked strategic tendencies and emotions associated with pleasure and frustration, whereas defensive focus increases sensitivity to negative outcomes by utilizing a “vigilance” strategy approach to ensure rejections. On the other hand, the defensive focus increases sensitivity to negative outcomes and employs a “vigilance” strategy approach to ensure rejection and avoid the strategic proclivity to do wrong, as well as emotions associated with calmness and excitement. A significant contribution of regulatory focus theory is the identification and differentiation of the various strategic means by which individuals pursue their objectives.

Further results obtained using the message framing can also be explained by the regulatory fit theory. Regulatory fit occurs when the strategic means used to accomplish a goal align with the individual’s regulatory focus, and this alignment increases the individual’s sense of value for the task at hand.

## 6. Policy Recommendation

While artificial meat has garnered much attention as an environmentally friendly, healthful, and safe substitute for meat, little study has been conducted on consumer attitudes toward artificial meat, particularly the types of messages utilized to communicate with consumers. Our analysis of nationally representative online survey data is thus designed to shed light not only on the consumer attitude to artificial meat but also on the extent to which the attitude is moderated by message framing.

These findings, which echo those of previous research, have important implications for policy. Above all, they underscore the urgent need to understand how to communicate with consumers. Industry and policymakers who are concerned with food marketing and health communication should employ different communication strategies depending on the type of consumer; for example, for consumers that use promotion focus, use gain-oriented messages. Different communication methods are used depending on the type of product. Avoidance-oriented messages, for example, are better for cultured meats.

## Figures and Tables

**Table 1:** Balance check.

Characteristic	Neutral Message Group (838)	Gain-Oriented Message Group (692)	Avoidance-Oriented Message Group (736)	F
Meat belief—health	3.980 (0.684)	3.950 (0.692)	3.953 (0.713)	0.43
Meat belief—animal welfare	3.060 (0.761)	3.103 (0.764)	3.105 (0.786)	0.88
Meat belief—environment	3.113 (0.605)	3.097 (0.638)	3.127 (0.644)	0.41
Meat belief—enjoyment	3.968 (0.694)	3.985 (0.686)	3.981 (0.669)	0.13
Consider price	3.862 (0.878)	3.902 (0.848)	3.887 (0.894)	0.42
Consider flavor	4.195 (0.807)	4.197 (0.822)	4.136 (0.819)	1.33
Consider health	4.229 (0.960)	4.233 (0.939)	4.189 (0.915)	0.50
Consider environment	3.187 (1.009)	3.182 (1.069)	3.110 (1.041)	1.30
Consider animal welfare	2.479 (1.036)	2.539 (1.040)	2.447 (1.065)	1.42
Food involvement	3.273 (0.654)	3.253 (0.666)	3.307 (0.627)	1.26
Food tech attitude	2.642 (0.754)	2.674 (0.756)	2.697 (0.741)	1.07
Food neophobia	2.771 (0.818)	2.757 (0.794)	2.742 (0.819)	0.25

Standard errors in parentheses.

**Table 2:** The structural model of plant-based meat (standardized coefficients).

	Benefit	Risk	Attitude
**Panel A:** Neutral message group			
Pro	0.279 ***	−0.239 ***	
	(0.0292)	(0.0357)	
Pre	−0.123 ***	0.147 ***	
	(0.0253)	(0.0162)	
Benefit			0.588 ***
			(0.0347)
Risk			−0.426 ***
			(0.0313)
**Panel B:** Gain-oriented message group			
Pro	0.561 ***	−0.475 ***	
	(0.0203)	(0.0198)	
Pre	−0.113 ***	0.108 ***	
	(0.0107)	(0.0113)	
Benefit			0.421 ***
			(0.0407)
Risk			−0.518 ***
			(0.0337)
**Panel C:** Avoidance-oriented message group			
Pro	0.286 ***	−0.263 ***	
	(0.0399)	(0.0401)	
Pre	−0.0924 ***	−0.0843 ***	
	(0.0211)	(0.0227)	
Benefit			0.430 ***
			(0.0383)
Risk			−0.503 ***
			(0.0331)

Standard errors in parentheses. *** *p* < 0.01.

**Table 3:** The structural model of cultured meat (standardized coefficients).

	Benefit	Risk	Attitude
**Panel A:** Neutral message group			
Pro	0.163 ***	−0.145 ***	
	(0.00908)	(0.0145)	
Pre	−0.289 ***	0.255 ***	
	(0.0509)	(0.0423)	
Benefit			0.583 ***
			(0.0284)
Risk			−0.493 ***
			(0.0268)
**Panel B:** Gain-oriented message group			
Pro	0.211 ***	−0.218 ***	
	(0.0152)	(0.0356)	
Pre	−0.231 ***	0.233 ***	
	(0.0587)	(0.0590)	
Benefit			0.576 ***
			(0.0321)
Risk			−0.549 ***
			(0.029)
**Panel C:** Avoidance-oriented message group			
Pro	0.141 ***	−0.103 ***	
	(0.0318)	(0.0218)	
Pre	−0.0611 ***	0.0739 ***	
	(0.0237)	(0.0216)	
Benefit			0.404 ***
			(0.0346)
Risk			−0.541 ***
			(0.0294)

Standard errors in parentheses. *** *p* < 0.01.

**Table 4:** Group-level goodness of fit.

	Plant-Based Meat	Cultured Meat
SRMR	0.091	0.093
CD	0.940	0.963

## Data Availability

Not applicable.

## Appendix A

**Table A1:** Messages presented in control and treatment information groups.

Information Group	Message
Control group participants: 838	Artificial meat products are primarily made from two food technologies: plant-based meat and clean meat. Plant-based meat is an alternative for regular meat which contains ingredients such as proteins, oils, and starches. For consumers, plant-based meat is available on market. Clean meat is an alternative for regular meat which is also known as lab-grown, in vitro, or cultured meat. It is the meat that is grown in cell culture rather than an animal’s body. For consumers, the clean meat products of Mosa Meat are expected to be on market in 2021.
Treatment group: Gain-oriented Message participants: 692	Control group message plus: Plant-based meat is produced with natural plant-based ingredients, which is an environmentally friendly substitute for regular meat. The clean meat is produced in a natural way similar to the fermentation process of yogurt or beer production, which has been used for the last thousand years in human history. Artificial meat is an eco-friendly, sustainable, and safe food. It supplements nutrition with ingredients such as unsaturated fatty acid, which improves health. Compared to regular meat products, artificial meat products are green, healthy, and nutritious, and significantly improves animal welfares.
Treatment group: Avoidance-oriented Message participants: 736	Control group message plus: The traditional meat production inevitably involves large-scale animal farming and slaughter. The greenhouse gas emission from the husbandry sector is beyond the total emission from the transportation sector. Half of world’s antibiotics are used during meat production and animal plagues happen occasionally. Therefore, traditional meat production is neither eco-friendly nor free of safety problems. Compared to regular meat products, artificial meat products can avoid environmental destruction, animal slaughter, and food safety problems.

**Table A2:** Frequency Distribution of Respondents’ Characteristics.

Variable	Description	Frequency	Percentage
Gender	Female	1091	48.15
	Male	1175	51.85
Education	Middle school and below	15	0.66
	High school	79	3.49
	Some college	310	13.68
	Bachelor’s degree	1661	73.30
	Master’s degree	189	8.34
	Ph.D. degree or equivalent	12	0.53
Monthly Income	Under 2000 RMB	266	11.74
	2000–4000 RMB	261	11.52
	4000–6000 RMB	488	21.54
	6000–8000 RMB	484	21.36
	8000–10,000 RMB	385	16.99
	10,000–20,000 RMB	319	14.08
	Above 20,000 RMB	63	2.78
Age	Under 18	15	0.66
	18–25	674	29.74
	26–30	624	27.54
	31–40	729	32.17
	41–50	177	7.81
	51–60	40	1.77
	Above 60	7	0.31

**Table A3:** Variable measurement.

Variable	Indicator	Description	Mean	SD
Promotion Focus	Pro1	I usually think about how to achieve expectations and goals	4.006	0.702
	Pro2	I will care about how to achieve success	3.918	0.839
	Pro3	I usually think good things relate to me	3.526	0.946
	Pro4	I care more about positive aspects of life	3.235	1.095
Prevention Focus	Pre1	I usually think about how to avoid failure	2.373	0.949
	Pre2	I usually worry about that I didn’t try my best	3.788	0.857
	Pre3	I usually worry about things that I fear to happen	2.650	1.202
	Pre4	I tend to avoid negative aspects of life	3.402	1.059
Food Neophobia	FN1	I always eat all kinds of novel food *	3.063	1.094
	FN2	I don’t trust novel food	2.549	1.000
	FN3	I like to try the food which I am not familiar with *	2.832	1.096
	FN4	I am afraid of the food which I have never eaten before	2.584	1.196
Food Involvement	FI1	I don’t spend much time thinking about the food I eat *	3.231	1.090
	FI2	I like to talk about the food I have eaten or will eat	3.774	0.891
	FI3	What to eat is not important to me comparing to other choices in life *	3.238	1.160
	FI4	I am familiar with nutrition facts of food	3.000	1.038
	FI5	My friends take my food recommendation seriously	3.145	1.075
Food Technology Attitudes	FT1	Support Genetically Modified Food technology	2.619	1.134
	FT2	Support Food additives	2.360	1.008
	FT3	Support Nano food technology	3.032	0.926
Meat Consumption Behavior	MCB1	Consider price	3.882	0.874
	MCB2	Consider flavor	4.176	0.816
	MCB3	Consider health	4.217	0.939
	MCB4	Consider environment	3.161	1.038
	MCB5	Consider animal welfare	2.487	1.047
Meat Belief—Enjoyment	MB1	Meat is delicious	4.233	0.755
	MB2	Meals with meat yield worse flavor *	3.957	1.027
	MB3	I eat meat almost everyday	3.701	1.084
	MB4	Meals with meat yield better flavor	3.998	0.853
Meat Belief—Health	MB5	Eating meat is not good for health *	3.756	0.969
	MB6	The nutrition facts in meat are important to health	4.168	0.738
Meat Belief—Environment	MB7	Meat production is harmless to environment	3.278	0.956
	MB8	Meat production increases greenhouse gas *	2.947	1.005
Meat Belief—Animal welfare	MB9	Animal killing for food is reasonable	3.276	1.037
	MB10	Animal welfare is important *	2.899	0.982
attitudes toward plant-based meat	A1	Plant-based meat is expected to be a major trend in the future.	3.620	0.909
	A2	I am receptive to technologies based on plant-based meat.	3.708	0.931
	A3	I’m curious to see how plant-based meat tastes.	3.883	0.904
	A4	I’d like to substitute plant-based meat for regular meat.	3.110	1.072
attitudes toward cultured meat	A1	cultured meat is expected to be a major trend in the future.	3.606	0.934
	A2	I am receptive to technologies based on cultured meat.	3.570	0.979
	A3	I’m curious to see how cultured meat tastes.	3.673	0.982
	A4	I’d like to substitute cultured meat for regular meat.	3.008	1.131
Perceived benefit of plant-based meat	B1	I believe that plant-based meat can help protect animal welfare.	4.088	0.880
	B2	I believe that plant-based meat can help reduce greenhouse gas emissions.	3.869	0.912
	B3	I believe that plant meat does not require antibiotics, veterinary drugs, or hormones.	3.774	1.088
	B4	I believe that plant-based meat has the potential to improve nutrition and is healthier for the human body.	3.839	1.072
Perceived benefit of cultured meat	B1	I believe that cultured meat can help protect animal welfare.	3.977	0.948
	B2	I believe that cultured meat can help reduce greenhouse gas emissions.	3.765	0.967
	B3	I believe that cultured meat does not require antibiotics, veterinary drugs, or hormones.	3.554	1.148
	B4	I believe that cultured meat has the potential to improve nutrition and is healthier for the human body.	3.481	1.148
Perceived risk of plant-based meat	R1	I feel the color, smell and taste of the plant-based meat is bad	3.042	0.971
	R2	I believe that plant-based meat is not healthy.	2.289	1.035
	R3	I believe that plant-based meat is not safety.	2.823	1.246
	R4	I believe that plant-based meat is not natural.	2.549	1.097
Perceived risk of cultured meat	R1	I feel the color, smell and taste of the cultured meat is bad	2.879	1.004
	R2	I believe that cultured meat is not healthy.	2.676	1.076
	R3	I believe that cultured meat is not safety	3.135	1.232
	R4	I believe that cultured meat is not natural.	2.846	1.109

Note: The values of variables Pro1–Pro4, Pre1–Pre4, FN1–FN4, FI1–FI5, and MB1–MB10 range from 1–5 (1 = totally not agree, 2 = not agree, 3 = neutral, 4 = agree, 5 = strongly agree); values of variables FT1–FT3 range from 1–5 (1 = never, 2 = not support, 3 = neutral, 4 = support, 5 = strongly support); values of variables MCB1-MCB5 range from 1–5 (1 = never, 2 = seldom consider, 3 = occasionally consider, 4 = usually consider, 5 = always consider). “*” denotes that such variables yield reverse score, for example, if someone totally not agree “I always eat all kinds of novel food”, the score of this question is 5 instead of 1.

**Table A4:** Frequency distributions of respondents’ acceptance toward artificial meat across information groups.

Artificial Meat	Variables	Totally Not Agree	Not Agree	Neutral	Agree	Strongly Agree
Panel A Control group (Neutral information group, 1257 participants)						
Plant-based meat	Plant-based meat is expected to be a major trend in the future.	2.15	10.26	32.34	41.17	14.08
	I am receptive to technologies based on plant-based meat.	2.15	9.19	28.16	43.44	17.06
	I’m curious to see how plant-based meat tastes.	2.86	5.97	19.93	49.28	21.96
	I’d like to substitute plant-based meat for regular meat.	8.00	25.30	36.63.	21.72	8.72
Clean meat	cultured meat is expected to be a major trend in the future.	2.63	12.29	30.43	39.74	14.92
	I am receptive to technologies based on cultured meat.	2.74	13.13	30.55	37.83	15.75
	I’m curious to see how cultured meat tastes.	3.46	9.90	25.66	42.72	18.26
	I’d like to substitute cultured meat for regular meat.	12.89	25.06	33.41	19.93	8.71
Panel B Treatment group I (gain-oriented information group, 1064 participants)						
Plant-based meat	Plant-based meat is a future trend	1.30	8.67	26.16	46.53	17.34
	I accept plant-based meat technologies	1.16	6.79	26.45	44.94	20.66
	I would like to try plant-based meat	1.16	5.49	15.75	51.73	25.87
	I would like to eat plant-based meat	6.94	17.49	34.97	29.62	10.98
	instead of regular meat					
Clean meat	Clean meat is a future trend	2.02	8.82	28.61	41.91	18.64
	I accept clean meat technologies	2.31	10.40	30.20	40.90	16.18
	I would like to try clean meat	2.60	7.95	23.27	44.51	21.68
	I would like to eat clean meat	6.94	23.41	32.66	25.43	11.56
	instead of regular meat					
Panel C Treatment group II (avoidance-oriented information group, 1120 participants)						
Plant-based meat	Plant-based meat is a future trend	1.22	9.92	27.85	47.01	13.99
	I accept plant-based meat technologies	1.63	9.24	25.27	42.66	21.20
	I would like to try plant-based meat	1.49	5.43	21.20	46.33	25.54
	I would like to eat plant-based meat	6.52	18.48	36.96	26.36	11.68
	instead of regular meat					
Clean meat	Clean meat is a future trend	1.49	7.07	30.84	46.06	14.54
	I accept clean meat technologies	2.04	10.87	27.99	40.22	18.89
	I would like to try clean meat	2.72	9.10	25.82	44.29	18.07
	I would like to eat clean meat	8.83	22.55	33.02	23.91	11.68
	instead of regular meat					

Note: Figures in the table above are percentage values.
